# Androgen insensitivity syndrome: a review

**DOI:** 10.20945/2359-3997000000031

**Published:** 2018-03-23

**Authors:** Rafael Loch Batista, Elaine M. Frade Costa, Andresa de Santi Rodrigues, Nathalia Lisboa Gomes, José Antonio Faria, Mirian Y. Nishi, Ivo Jorge Prado Arnhold, Sorahia Domenice, Berenice Bilharinho de Mendonca

**Affiliations:** 1 Universidade de São Paulo Universidade de São Paulo Faculdade de Medicina, Hospital das Clínicas Unidade de Endocrinologia do Desenvolvimento, Laboratório de Hormônios e Genética Molecular São Paulo SP Brasil Unidade de Endocrinologia do Desenvolvimento, Laboratório de Hormônios e Genética Molecular/LIM42, Hospital das Clínicas, Disciplina de Endocrinologia, Faculdade de Medicina da Universidade de São Paulo (FMUSP), São Paulo, SP, Brasil; 2 Universidade de São Paulo Universidade de São Paulo Faculdade de Medicina Laboratório de Sequenciamento em Larga Escala São Paulo SP Brasil Laboratório de Sequenciamento em Larga Escala (SELA), Faculdade de Medicina da Universidade de São Paulo (FMUSP), São Paulo, SP, Brasil

**Keywords:** Androgen insensitivity syndrome, androgen receptor, disorders of sex development, 46,XY DSD

## Abstract

Androgenic insensitivity syndrome is the most common cause of disorders of sexual differentiation in 46,XY individuals. It results from alterations in the androgen receptor gene, leading to a frame of hormonal resistance, which may present clinically under 3 phenotypes: complete (CAIS), partial (PAIS) or mild (MAIS). The androgen receptor gene has 8 exons and 3 domains, and allelic variants in this gene occur in all domains and exons, regardless of phenotype, providing a poor genotype – phenotype correlation in this syndrome. Typically, laboratory diagnosis is made through elevated levels of LH and testosterone, with little or no virilization. Treatment depends on the phenotype and social sex of the individual. Open issues in the management of androgen insensitivity syndromes includes decisions on sex assignment, timing of gonadectomy, fertility, physcological outcomes and genetic counseling.

## INTRODUCTION

Androgen Insensitivity Syndrome (AIS) is an X-linked genetic disease and it is the most common cause of disorders of sex development (DSD) in 46,XY individuals (1). The phenotype ranges from normal female external genitalia in the complete form (CAIS) to normal male external genitalia associated with infertility and/or gynecomastia in the mild form (MAIS). A large spectrum of undervirilized male external genitalia is observed in the partial form (PAIS) (2). Mutations in the androgen receptor gene (*AR*) are found in most individuals with CAIS but in less individuals with PAIS (3).

AIS was first described by Morris, in 1953, with the clinical description of 82 female patients with testes but female phenotype and for this reason Morris named the syndrome as testicular feminization (4). Later, this syndrome was characterized for being a condition resulting from a complete or partial resistance to androgens in 46,XY individuals with normal male gonad development (5).

PAIS should be considered in all individuals with atypical genitalia at birth regardless of the degree of external genitalia virilization and MAIS is a possible diagnosis in males with persistent gynecomastia and or infertility (6).

Role of Androgens in Male Fetal Development: androgens are key elements for appropriate internal and external male sex differentiation. After normal testes development, the Leydig cells produce testosterone, which promotes Wolffian duct differentiation into epididymes, vasa deferentia and seminal vesicles (7). The conversion of testosterone to dihydrotestosterone by the 5α-reductase type 2 enzyme promotes male external genitalia differentiation (8). In humans, the critical period for genitalia virilization occurs between 8 and 14 weeks of gestation and depends on the presence of androgens and of a functioning androgen receptor (9). Impairment of androgen secretion and defects in the androgen receptor will compromise the virilization process.

## THE HUMAN ANDROGEN RECEPTOR

**Figure 1:**
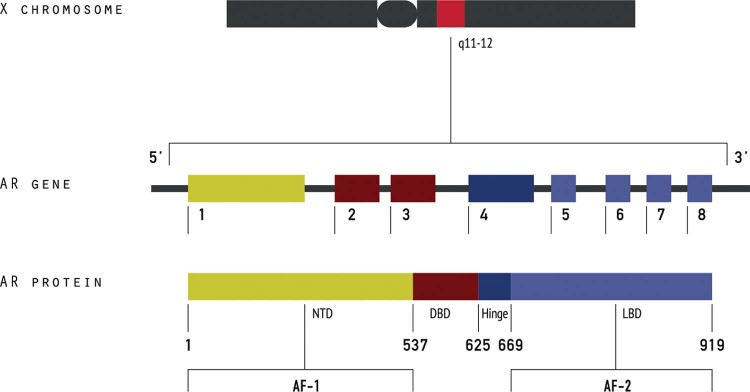
A schematic representation of androgen receptor gene and androgen receptor protein.

The *AR* gene is located at chromosome Xq11-12, is encoded by eight exons and codifies a 919 aminoacids protein (Figure 1). The *AR* is a ligand-dependent transcription factor composed by three functional domains as the other nuclear receptors: a large N-terminal domain (NTD) (residues 1-555), a DNA-binding domain (DBD) (556-623 residues), a hinge domain (624-665 residues) and a C-terminal ligand-binding domain (LBD) (666–919 residues) (10). The NTD is encoded by exon 1 and contains a ligand-independent transactivation function 1 (AF1), which contains two distinct transcription activation units: Tau-1 (aminoacids 100-370) and Tau-5 (aminoacids 360-485), that are essential for full *AR* activity. The DBD is composed by two zinc fingers and connects the *AR* to promoter and enhancer regions of *AR* regulated genes by direct nuclear DNA binding allowing the activate functions of NTD and LBD (11). The LBD is encoded by exons 4-8 and contains 11 α-helices associated with two anti-parallel β-sheets in a sandwich-like conformation with a central ligand binding pocket, in which the ligand can bind (12).

## CLINICAL PRESENTATION

CAIS prevalence in 46,XY males is estimated from 1 in 20.400 to 1 in 99.100 (13). Except in cases of familial inheritance, CAIS is diagnosed in three scenarios: in fetal life when prenatal sex determination disclosed a 46,XY karyotype in a fetus with female external genitalia; in childhood in a girl with inguinal hernia or at puberty in females with primary amenorrhea (14). The presence of inguinal hernia in a female child is rare and could indicate a CAIS diagnosis (13). Patients with AIS developed breasts with estradiol levels in normal male range suggesting that the lack of androgen action is the main driver of breast development in these patients, rather than an increased estrogen secretion. Menstrual cycles do not appear since normal production of anti-mullerian hormone (AMH) by the testis impeded uterus, cervix and proximal vagina to development. A shortened blind-ending vagina is observed in almost all patients and the vaginal measurement varied from 2.5 to 8 cm in CAIS and 1.5 – 4 cm in PAIS. Pubic and axillary hair are sparse or absent (1,14).

Final height in CAIS is above normal mean female height, probably due to the action of the growth-controlling gene (GCY) located at the Y chromosome (15). Interestingly, newborns with CAIS have the same size of male newborns, suggesting that postnatal factors are involved in the final height in these individuals (16). In our cohort, the final height of CAIS individuals (165.7 ± 8.9 cm) was taller than described for Brazilian females, but lower than expected for Brazilian males (15).

Differential diagnosis of CAIS includes complete gonadal dysgenesis, Mayer-Rokitanski-Kuster-Hauser syndrome and Mullerian ducts anomalies (1). Biosynthetic enzyme deficiencies are rarely a differential diagnosis for CAIS (8,17).

The PAIS clinical phenotype varies according to the degree of *AR* residual function and ranges from proximal hypospadias to micropenis (18). Hypospadias are a common finding with an estimated prevalence of 1:8000 male births and *AR* sequencing is necessary to exclude PAIS diagnosis (19). Gynecomastia observed at puberty time in patients with atypical genitalia can be indicative of PAIS (2,20). Differential diagnosis of PAIS includes all causes resulting in a undervirilized male external genitalia such as chromosomal defects (Klinefelter syndrome), genetic diseases (Smith-Lemli-Opitz syndrome, Denys-Drash syndrome, Frasier syndrome), partial gonadal dysgenesis, LH receptor defects, biosynthetic enzyme deficiencies (17,20-lyase deficiency, P450 oxidoreductase deficiency, 17β-hydroxysteroid dehydrogenase deficiency type 3, 5α-reductase 2 deficiency and hypospadias in small for gestation age boys (8,17).

MAIS is associated with *AR* mutations but without external genitalia abnormalities (6). This diagnosis could be suspected in the investigation of male infertility or in pubertal gynecomastia (14,18). There are few *AR* mutations associated exclusively with MAIS, but this condition is probably underdiagnosed (3,6).

MAIS can also manifest in a patient with neurological disorder characterized by bulbar and muscular atrophy (Kennedy's disease). This condition is due to the hyperexpansion of the CAG repeats (> 38), present in *AR* exon 1 (21). These patients present with normal male external genitalia, but testosterone resistance will develop with disease progression. For MAIS, the differential diagnosis includes other causes of male infertility.

## ENDOCRINE FEATURES

In AIS the endocrine profile is consistent with androgen resistance characterized by elevated or normal basal serum testosterone levels associated with high serum LH levels (22). Elevated serum AMH and testosterone levels in a newborn suggest the diagnosis of androgen insensitivity and also exclude the diagnosis of complete gonadal dysgenesis (23). In postpuberal patients estradiol levels are normal or slightly elevated for a male individual (22). This pattern is seen at mini-puberty or after puberty. During childhood when gonadotropin axis is not activated, a hCG stimulation is necessary to evaluate testosterone secretion by Leydig cells (24). In MAIS, hormone concentrations are usually normal, but elevated serum LH and testosterone levels could be found in these patients (19).

Typically in AIS, basal testosterone and LH levels are elevated demonstrating the impairment of androgen negative feedback on the anterior pituitary (22). In contrast, FSH levels are usually normal in AIS. This is explained by the fact that FSH is mainly regulated by gonadal inhibin (25). Although there are differences in the *AR* residual function among the mutated receptors between CAIS and PAIS phenotypes, no difference are observed in hormonal levels (20,22). Serum LH, FSH estradiol, DHT were not different in subjects with CAIS and PAIS (Table 1).

**Table 1:** Basal hormone levels in patients with AIS

Phenotype	LH (U/L)	FSH (U/L)	Testosterone ng/dL	Estradiol pg/mL	Reference
CAIS	14 – 43*	3.5 – 16*	186 – 1033*	10 – 40*	(22)
n = 11	26**	7.4**	342**	27**	
PAIS	9 – 32*	– 34*	157 – 1592*	20 – 109*	(22)
n = 14	26**	5.0**	1032	49	
CAIS	5.5 – 51	0.4 – 16**	173 – 1497*	4.8 – 70*	(60)
n = 42	18.5	3.5*	576**	30.7**	

* Range;

** Median.

## MOLECULAR DEFECTS IN THE ANDROGEN RECEPTOR GENE

The AIS diagnosis is confirmed by the presence of allelic variants in the *AR* gene (1,26). About 30% of *AR* mutations in AIS are *de novo* and sequencing of the entire *AR* gene is recommended for all 46,XY DSD newborns, regardless of a familial history of DSD or AIS (26). In the absence of allelic variants in *AR* a multiplex ligation-dependent probe amplification (MLPA) can be helpful in order to detect deletions, insertions and duplications in the *AR* gene (26). There are more than 1000 *AR* mutations described in a website database associated with AIS and prostate cancer (http://www.mcgill.ca/androgendb) and around 600 of them were described in AIS (3). Mutations are found along the *AR* gene, being more frequent in exon 1 (the largest *AR* exon, which encodes the NTD). Defects in the NTD domain are more frequent in CAIS's patients and variants in exons 5 and 6 (that encode LBD) are more frequent in PAIS's patients (3). Almost all *AR* mutations in MAIS were found in the NTD, but there is a low number of *AR* mutations related to this phenotype.

The most common *AR* allelic variants in all AIS phenotypes are non-synonymous point mutations. Insertions and deletions causing a frameshift leading to a premature stop codon downstream are more frequently reported in CAIS's patients. Allelic variants affecting mRNA splicing are reported in CAIS and PAIS phenotypes. Rarely, synonymous allelic variants affecting splicing sites has been described in PAIS (27) and in CAIS individuals (28).

Large structural mutations (exon 1 deletion, exon 2 duplication, exon 3 deletion, exon 4-8 (LBD domain) deletion and deletion of entire *AR* gene) have been described but are very rare in AIS (3). Interesting, a deletion of an entire exon (exon 4) was previously described in a phenotypic male with azoospermia (29).

Postzygotic *AR* allelic variants resulting in somatic mosaicism are rarely described in AIS (30). In this situation the variant appears in heterozygote instead of hemizygote state. *AR* allelic variants in heterozygosis was also identified in some individuals with 47,XXY karyotype causing AIS (31).

There is not a perfect correlation between genotype and phenotype in AIS. In the *AR* mutation database, there are some *AR* allelic variants that can cause different phenotypes (Table 2). The explanation for this is not completely understood. It is hypothesized that *AR* co-regulators (activators and repressors) are implicated with this phenomenon. Other possibilities are variations in the level of 5α-reductase type 2 activity resulting in different DHT availability, and the presence of germ-line *AR* allelic variants at a post zygote stage conferring somatic mosaicism (31).

**Table 2:** *AR* allelic variants identified in more than one AIS phenotype (3)

Allelic variants	Phenotype
p. Leu174, p. Arg616Pro, p. Asn693del, p. Asn706Ser,p. Gly744Val, p. Met746Phe, p. Met750Val, p. Trp752*, p. Ala766Thr, p. Pro767Ser, p. Arg775His, p. Arg841His, p. Ile843Thr, p. Val867Met, p. Val890Met, p. Ser704Gly	CAIS, PAIS
p. Pro392Ser, p. Leu548Phe, p. Arg616His, p. Asp696Asn, p. Met781Ile, p. Arg856His, p. Ala646Asp	CAIS, PAIS, MAIS
p. Tyr572His, p. Arg608Gly, p. Asn757Ser, p. Arg789Ser, p. Gln799Glu, p. Thr801Ile, p. Ser815Asn, p. Leu822Val, p. Ala871Gly, p. Gly216Arg, p. Arg608Gly	PAIS, MAIS

## CLINICAL MANAGEMENT OF AIS

AIS patients have complex issues including functional, sexual and psychosocial aspects. Sex assignment, external genitalia adequacy for social sex, hormonal replacement, psychosexual outcome, ideal time for gonadectomy, infertility and genetic counseling are issues that need attention in AIS care. All of them demand flexible, sensible and individualized procedures to achieve good results.

## CLINICAL MANAGEMENT OF CAIS

After diagnosis, the first aspect to be considered is the time for bilateral gonadectomy. In a girl, maintenance of the gonads will allow spontaneous breast development, though breast development is similar with estrogen replacement in gonadectomized females. So far, gonadectomy is performed at early age, in order to avoid the risk of malignancies and the psychosocial difficulties in submitting an adolescent female to gonadectomy (24). When gonadectomy is performed before puberty, estrogen replacement is necessary to induce puberty. In general, hormonal replacement is started at the age of 11-12 years with oral or transdermal estrogen. Both ways are adequate and the patient and family can choose the route in which the compliance will be better (18). Due to the absence of uterus, progesterone replacement is not necessary.

Genitoplasty is not necessary in CAIS and vaginal dilation promotes an adequate vaginal length vaginal dilation should occur after puberty or when the patient refers to desire to initiate sexual activity (32). Most of the individuals (80%) who were submitted to vaginal dilation referred satisfactory and some of them reported dyspareunia (33). There are many vaginoplasty techniques (34), but non-surgical dilation is effective, safe, non expensive and normalizes vaginal length and sex intercourse (32). Because of that, surgical creation of a vagina should be avoid regardless of the surgical technique (32).

## CLINICAL MANAGEMENT OF PAIS

PAIS diagnosis is usually suspected in a newborn with atypical genitalia and palpable gonads. Most of the patients are raised as male. The degree of external genitalia virilization is related to the residual AR function and can be predictive of androgen response at puberty. In male patients, correction of cryptorchidism and hypospadias are recommended as soon as possible, preferably before two years of age (35).

PAIS males frequently develop gynecomastia at puberty and surgical correction is generally necessary (22). High testosterone or DHT trials (intramuscular or topic testosterone esters or topic DHT) can be use to increase penile length and to improve other virilization signs (18,30). The results are unpredictable but are usually limited. Maximum virilization effect is observed after 6 months of high androgen usage treatment, subsequently, androgen therapy can be withdrawn in the patients with normal testes and preserved testosterone secretion.

For individuals raised as females, bilateral gonadectomy is recommended in childhood to avoid virilization and to eliminate the risk of testicular tumors (36). Genitoplasty is usually necessary in PAIS females and estrogen replacement is mandatory at pubertal time, with similar recommendation as describe for CAIS patients (15).

For MAIS, there is little information about clinical outcomes. Gynecomastia and infertility are the usual clinical presentation of this phenotype (6) and mastectomy is recommended for gynecomastia correction. This phenotype is observed in individuals with Kennedy's disease, which is more commonly known as spinal and bulbar muscular atrophy (SBMA). This syndrome is caused by an excessive number of CAG repeats in the *AR* exon 1 and a number of patients also have testicular atrophy, gynecomastia, oligospermia and erectile dysfunction (37).[Table t3]

**Table 3 t3:** Types of androgen receptor allelic variants related to AIS reported in the androgen receptor mutations database

Type of defect	CAIS	PAIS	MAIS
Non-synonymous	155	125	41
Stop codon	57	2	0
Indel	41	4	2
Duplication	6	0	0
Total	259	131	43

## HORMONAL REPLACEMENT IN AIS

Hormonal replacement is mandatory for all gonadectomized individuals. In females, the purpose is the development of secondary sexual characteristics and an adequate and bone mass (2). Estrogen can be introduced in low doses (one quarter of the adult dose), at 9 – 11 years of age, with titration of this dosage every 6 months (20). The time for complete feminization is expected to be about 2 years. Oral or transdermic estrogen are alternative ways for estrogen replacement. The initial dose is 0.25 mg/day of 17β-estradiol increasing the dose each 6 months considering the progression of breast development. After complete breast development, a regular dose can be introduced (1-2 mg/day of 17β-estradiol continuously) (9).

In male individuals, the testes are able to produce testosterone. In male AIS, at pubertal age, high testosterone doses (200–500 mg twice a week) can be used, in order to increase the penile size and to promote virilization (1). Maximum penile length is obtained after six months of treatment with high testosterone doses. After this period, the dose of testosterone when necessary should return to the maintenance dose. The use of DHT in male PAIS has been tested (0.3 mg/kg of androstanolone gel 2.5% for 4 months) and mixed results were obtained following DHT therapy (38).

## GONADAL TUMOR RISK IN AIS

Disorders of sex development are recognized as a risk factor for type II germ cell tumors (GCTs). These tumors are classified as seminomatous and non-seminomatous types (39). The seminomatous tumors referred to seminoma (testis) and to dysgerminoma (ovary and dysgenetic gonads). In the non-seminomatous group, many differentiated variants can be identified according to the cellular origin, being the teratomas from somatic differentiation, yolk sac tumor and choriocarcinoma from extra-embryonic differentiation, and embryonal carcinoma from stem cells (27). These tumors derivate from a non-invasive precursor named carcinoma *in situ* – CIS – or Intrabular germ cell neoplasia unclassified – IGCNU). In 2016, the World Health Organization suggested to change the nomenclature of this initial germinative neoplastic lesion from CIS or IGCNU to germ cell neoplasia *in situ* (GCNIS) (40). GCNIS are always non-invasive, but 50% of GCNIS progress to invasive GCTs within 5 years. The risk of GCTs development is related to the presence of a Y chromosome, but is not the same for the different etiologies of 46,XY DSD. So far, some factors, as chronological age and gonadal location can influence GCTs development (41).

In CAIS, the risk of GCTs is considered low and related to age (36). The estimated risk of gonadal tumors in CAIS gonads was about 0.8% - 22% (42). However, most old series included patients without confirmed AR mutation or without description of age at gonadectomy. The reports of malignant GCTs before puberty in CAIS are very rare (43). There is only one documented report of an invasive yolk-sac tumor in a CAIS individual before puberty. This occurred in a 17-months-old CAIS girl with abdominal gonads (44). After puberty, the risk is low, but not negligible. In a study, including 133 patients with CAIS, the gonads' histological and immunohistochemical findings showed a prevalence of 1.5% (2/133) for malignancies (45). The low incidence of GCTs in CAIS individuals can be explain by the rapid decline of germ cells after the first year of life (46).

PAIS individuals may maintain their germ cells because of the presence of residual androgen receptor responsiveness, differently of CAIS (46). Therefore, the incidence of GCTs in PAIS (15%) is higher than in CAIS (42). In cases of PAIS with untreated undescended testes the GCTs risk may be as high as 50% (47). Therefore, laparoscopic bilateral gonadectomy is indicated in all PAIS females and orquidopexy in scrotum in the male patients (48).

In patients who maintained the gonads, a careful monitoring including ultrasonography (US) or MRI has been suggested (43). Due to easy access and low cost, US remain the first choice for monitoring retained gonads. MRI has demonstrated adequate sensitivity to detect benign gonadal lesions, such as cysts or Sertoli cell adenomas, but failed to detect GCNIS (49). Annual US follow-up of labioscrotal and/or inguinal gonads is recommended. For abdominal gonads monitoring MRI is more helpful (50).

## FERTILITY IN AIS

A normal androgen receptor is necessary for normal male reproduction, because testosterone and FSH, are essential factors for male spermatogenesis. Therefore, mutations in the androgen receptor gene have been searched in order to identify possible causes for male infertility. As previously described, infertility may be the only clinical manifestation of undervirilization in MAIS phenotype (6,51).

The strategy to obtain fertility in AIS individuals has not been defined yet (52). In CAIS, there is absence of uterus and testes histology reveals incomplete spermatogenesis, increased fibrosis, Leydig cell hyperplasia and low frequency of spermatogonia conferring a very low potential to fertility. In addition, the viability of male germ cells in CAIS is restricted to the first two years of life and for fertility in adult life germ cells should be preserved before this age (46). In PAIS individuals, some residual androgen receptor function is preserved, but not usually enough to promote fertility (46). Indeed, infertility is the rule in AIS (22).

Probably, fertility is the most sensitive outcome which depends of an intact androgen receptor. For it, MAIS individuals can present only infertility (6,51). However, the p.G824K and p.R840C *AR* variant allelics, were found in male individuals with preserved fertility (51,53).

A successful fertility was recently described in a PAIS individual harboring the p.V686A *AR* variant, after prolonged high-dose testosterone therapy (250 mg of testosterone enanthate weekly by four years) causing improvement in sperm count. The gonadotropin concentrations remained unaffected and intracytoplasmic sperm injection with a single sperm directly into an egg resulted in proved fertility (54).

In general, infertility in AIS is the rule. The evidence of sperm count improvement after high doses of testosterone (as described above) can be an indicative of fertility success, but should be tested in further studies as well as the use of aromatase inhibitors and clomiphene citrate to obtain fertility in these patients

## PSYCHOLOGICAL OUTCOMES

Psychological support is essential for AIS individuals and their parents, in general (55). Dialogue about fertility, sexuality and karyotype are delicated issues to be approached with AIS individuals.

The gender identity, gender role and sexual orientation show a female pattern in CAIS individuals. In PAIS patients, in general, gender identity aligned with both sex of rearing male or female (56).

Gender change is very rarely described in CAIS and there are just four cases of gender change in individuals with CAIS (57). Therefore, gender dysphoria in CAIS is considered truly transgenderism. However, sexual functioning and sexual quality of life demonstrated less-positive outcome in CAIS patients in comparison with normal woman (58).

Although there is no inconsistency in gender identity, male PAIS individuals show disappointment with undervirilization signs. The absence or paucity of facial and body hair, the high-pitched voice compromised their self-perception of manhood (59). In female individuals, low scores in feminility scales have been reported (58). An impairment of sexual functioning is reported in male and female PAIS individuals (58).

## CONCLUSION

AIS is the most common molecular diagnosis in newborns with 46,XY DSD and results of an *AR* defect. It has an X-linked inheritance and affects 50% of the male offspring. In CAIS, the diagnosis can be done intrauterus, at birth, childhood or after puberty. In PAIS, the diagnosis is usually at birth due to the atypical external genitalia. In MAIS, the diagnosis should be considered in cases of pubertal gynecomastia and male infertility. *AR* defects are found along *AR* gene in all AIS phenotypes. Non-synonymous point mutations are the commonest *AR* defects reported in AIS. Molecular diagnosis is achieved in almost all patients with CAIS and in a lower frequency in PAIS individuals. AIS is characterized by elevated serum LH and testosterone. In CAIS, there is a low risk of GCTs before puberty and postponing surgery to after puberty may allow the development of spontaneous puberty. In PAIS there is a risk of GCTs in 15% of the patients, and bilateral gonadectomy is recommended at childhood in all individuals raised in the female social sex. For males with PAIS, the testis should be placed in the scrotum and regularly monitored. Fertility was described in one PAIS individuals, and therapeutic strategy for successful fertility could be experienced in PAIS and MAIS individuals. In AIS, gender identity usually follows the sex of rearing, but quality of sexual life, sexual functioning and quality of life can be slightly compromised and are important issues for keeping patients in psychological care.
